# Behavioral outcome among survivors of childhood brain tumor: a case control study

**DOI:** 10.1186/s12887-020-1951-3

**Published:** 2020-02-05

**Authors:** Hamidah Alias, Sasirekha Krisnan Morthy, Syed Zulkifli Syed Zakaria, Zulaiha Muda, Azmi Mohd Tamil

**Affiliations:** 1 Department of Pediatrics, UKM Medical Centre, Faculty of Medicine, The National University of Malaysia, Cheras, 56000 Kuala Lumpur, Malaysia; 2 Institut Pediatrik, General Hospital Kuala Lumpur, The National University of Malaysia, Kuala Lumpur, Malaysia; 3 Department of Public Health, Faculty of Medicine, The National University of Malaysia, Kuala Lumpur, Malaysia; 4 Hospital Tunku Azizah, Kuala Lumpur, Malaysia

**Keywords:** Child behavior checklist, Youth self Report, Brain tumor, Survivors, Behavioral outcome

## Abstract

**Background:**

Advances in the treatment of childhood brain tumors have significantly improved survival rates. With improved survival rates, long-term treatment-related toxicities have become important, and the resulting complications can affect patients’ emotion and behavior. This study aimed to 1) evaluate behavioral outcomes among survivors of childhood brain tumors, 2) compare behavioral outcomes among survivors of childhood brain tumors with survivors of childhood leukemia and healthy children, and 3) determine any demographic, disease, and/or treatment-related factors that could affect the behavioral outcomes of survivors of childhood brain tumors.

**Methods:**

A comparative cross-sectional study was conducted over a period of 1 year (June 1st, 2018–May 31st, 2019) in two tertiary referral centers in Kuala Lumpur, Malaysia. Thirty-eight survivors of childhood brain tumors aged 6 to 18 years old who had been off-treatment for at least 1 year and were in remission, 38 age- and gender-matched survivors of childhood leukemia who had been off-treatment for at least 1 year and were in remission, and 38 age- and gender-matched unrelated healthy children were recruited. The Child Behaviour Checklist (CBCL) parent report and Youth Self-Report (YSR) questionnaires were used to assess behavioral outcomes.

**Results:**

Survivors of childhood brain tumors showed statistically significantly worse behavioral outcomes than healthy children for social problems and attention problems (*p* < 0.05, respectively). A significantly worse outcome was found for “social problems” (*p* < 0.05) in survivors of childhood brain tumors compared to survivors of childhood leukemia. Significant associations were also found between physical disability, visual impairment, education level of survivors, and father’s occupation and behavioral outcomes among survivors of childhood brain tumors.

**Conclusions:**

Survivors of childhood brain tumors in our center showed poor behavioral outcomes for social problems and attention problems. Thus, effective psychosocial support interventions tailored to individual patients as soon as treatment is completed are important to prevent potentially debilitating emotional problems.

## Background

The incidence of childhood brain tumors has increased by approximately 50% between the year 1975 and 2000 [1]. Brain tumors are the most common type of solid tumor in children and are a major cause of death among all childhood cancers [2]. The Malaysian National Cancer Registry Report 2007–2011 stated that brain and nervous system tumors are the second most common childhood cancers among Malaysian children aged between 0 and 14 years old. The national incidence of childhood brain and central nervous system (CNS) tumors is 2 per 100,000 children [3]. Childhood brain tumors are more common in males, although this varies according to histologic type. Benign tumors are generally treated with surgical excision alone, whereas malignant tumors are treated with a combination of chemotherapy and/or radiotherapy postoperatively [4].

Advances in childhood brain tumor treatment have significantly improved survival; the five-year survival rate was almost 75% with the proportion varying by tumor type [5]. With improved survival rates, attention to disease and/or treatment-related long-term complications such as neurological impairments, cognitive dysfunction and growth, and endocrine disturbances has increased [4]. Previous studies reported that survivors of childhood brain tumors were at risk of emotional and behavioral problems such as depression within months of ceasing treatment and returning to the community [4, 6–10]. These behavioral and emotional problems appear to persist into early adulthood and beyond [6].

Childhood and adolescent behavior can be broadly classified as internalizing and externalizing based on their reactions to stressors. Internalizing behaviors are characterized by anxiety, somatization, and/or depression [11]. Externalizing behaviors are characterized by acting out, antisocial behavior, hostility, and aggression [11]. Schultz KA et al. reported that survivors of CNS tumors, leukemia, and neuroblastoma were at risk for poorer behavioral and social outcomes. Survivors of childhood CNS tumors were reported to have significant depression/anxiety, attention deficit, antisocial behavior, and reduced social competence [8]. Internalizing behavior problems were found to be more frequent in survivors, whereas externalizing behaviors were relatively rare based on the parent-reported Child Behaviour Checklist (CBCL) results [4, 9, 10]. Another study reported greater withdrawal and social and attention problems in survivors with a longer time elapsed between diagnosis and assessment [9, 10]. By contrast, Carpentieri SC et al. and Holmquist LA et al. reported that childhood brain tumor survivors did not have more behavioral problems than survivors of non-CNS tumors [6, 12].

Scarce literature reports on associations between behavioral outcomes in survivors of childhood brain tumors and socioeconomic status (SES). Kullgren KA et al. reported that survivors of brain tumors with a lower SES were at a greater risk of behavioral problems [13]. These findings support the importance of developing effective psychosocial support interventions tailored to individual patients that are made accessible from diagnosis and particularly immediately after treatment cessation [4, 7, 9]. Furthermore, many studies have reported promising outcomes in survivors following individual and group interventions [14–16].

There are few studies on behavioral problems in survivors of childhood cancer in developing countries. We believe it is important to perform a similar study in Malaysia because the results may differ from those reported in developed countries. Factors such as multi-ethnicity and cultural beliefs could contribute to different behavioral problems in survivors. This study aimed to evaluate behavioral outcomes in survivors of childhood brain tumors who were treated at two tertiary referral centers and to compare the behavioral outcomes with survivors of childhood leukemia and unrelated healthy children. We also studied any demographic-related and/or disease- or treatment-related factors that could be associated with behavioral outcomes.

## Methods

### Subjects

This was a multicenter, comparative cross-sectional study conducted in the Pediatric Hematology and Oncology Unit at the Department of Pediatrics, Universiti Kebangsaan Malaysia Medical Centre and the Hospital Tunku Azizah, Kuala Lumpur over a 1 year period from June 1st, 2018 until May 31st, 2019. All survivors of childhood brain tumors aged 6–18 years old who had completed treatment at least 1 year previously and remained in remission were eligible for this study. Survivors who did not complete treatment, has preexisting behavioral disorders, and parents or patients who did not understand English or Bahasa Malaysia (the national language of Malaysia) were excluded. The two comparative groups consisted of survivors of childhood leukemia and unrelated healthy children, matched for gender and age. Survivors of childhood leukemia with no other illnesses and who had been off-treatment for at least 1 year and remained in remission were recruited. Healthy children not related to the survivors were randomly selected and recruited for the study. All eligible subjects were approached by one investigator. Written informed consent was obtained from the parents prior to subject recruitment. Ethical approval was obtained from the Research Ethics Committee of the Universiti Kebangsaan Malaysia and the Medical Research and Ethics Committee, Ministry of Health prior to the study. Approval for using the original versions of the CBCL and YSR was obtained from the founder, Prof. Dr. Thomas M. Achehbach, Professor of Psychiatry and Psychology, University of Vermont. The questionnaire was translated from the original English version into Bahasa Malaysia and was reviewed by the members of the research team for content validity. It was pilot tested on 30 parents whom were then included in the study. The internal validation of the questionnaire in Bahasa Malaysia was 0.952 (Cronbach’s alpha).

### Measures

#### CBCL and YSR

The CBCL parent report is a widely used instrument that assesses social competence and behavioral problems of children 6–18 years of age [17]. The problem scale comprised of 113 questions that fall into three global scales; internalizing, externalizing and total behavioral problems. Eight subscales were assessed: ‘anxious/depressed’, ‘withdrawn/depressed’, ‘somatic complaints’, ‘social problems’. ‘thought problems’, ‘attention problems’, ‘ rule-breaking behavior’, and ‘aggressive behavior’. Parents answered the questions based on their children behavior for the past 6 months. Each question was scored on a 3-point scale (0 = not true, 1 = somewhat or sometimes true, 2 = very true or often true). Raw scores of these measures were transformed to standardized T scores with higher scores reflecting more behavioral difficulties.

For the 3 global scales; internalizing, externalizing and total behavioral problems, T scores between 60 and 63 reflect borderline clinical range behavioral problems whereas T scores more than 63 reflect clinical range behavioral problems. For the 8 subcales, T scores between 65 and 69 reflect borderline clinical range behavioral problems whereas T scores more than 69 reflect clinical range behavioral problems. The CBCL has been shown to have strong reliability and validity in clinical and normal population [11].

YSR which was derived from CBCL is a self-administered form designed for use in children and adolescents aged 11–18 years. The questionnaire consists of 112 items addressing a variety of social competence and behavioral problems. It has 3 global scales with 8 subscales and scoring system similar to CBCL [17].

The parents of patients between 6 and 10 years old completed the CBCL questionnaire. Conversely, for patients between 11 and 18 years, both the parents and the patients completed the CBCL and YSR questionnaires, respectively.

##### Statistical analysis

All statistical calculations were conducted using the Statistical Product for the Service Solution program version 20. Since the data were not normally distributed, non-parametric tests were used for the statistical analyses. The Kruskal–Wallis test was conducted to evaluate the differences in behavioral outcomes among the three groups of survivors. When indicated, post hoc analyses with the Mann–Whitney U test were then conducted to perform pairwise comparisons. To determine any demographic, disease, and/or treatment-related factors that could affect behavioral outcomes, the continuous data were analyzed using the Mann–Whitney U test for two comparison groups, whereas the Kruskal-Wallis test was used for more than two comparison groups. Spearman’s rank order correlation was used to analyze the correlation between CBCL parental reports and the YSR. Non-normally distributed continuous data were presented as the median and centile. Categorical data were analyzed using the chi-square test and presented as the frequency and percentage.

## Results

### Subjects

A total of 72 survivors of childhood brain tumors who were still being followed-up were identified from the database. Thirty-four of the 72 survivors were excluded because they did not fulfill the inclusion criteria for the following reasons: younger than 6 years old (*n* = 6), older than 18 years old (*n* = 11), did not complete treatment (*n* = 1), less than 1 year removed from treatment (n = 6), on palliative chemotherapy (*n* = 3), language barrier (*n* = 1), did not provide consent (*n* = 1), uncontactable (*n* = 3), and did not return the questionnaire (*n* = 2). Thirty-eight survivors of childhood brain tumors, 38 age- and gender-matched childhood leukemia survivors, and 38 age- and gender-matched unrelated healthy children were recruited for this study. The demographic and clinical characteristics of the survivors of childhood brain tumors are shown in Tables 1 and 2. All relapsed patients were treated. Thirty-two of the 38 survivors had received radiotherapy as a part of treatment. All survivors of childhood leukemia had acute lymphoblastic leukemia (ALL) except one who had acute myeloid leukemia. There were no significant differences in the demographic variables between the survivors of childhood brain tumors and the survivors of childhood leukemia and healthy children except for ethnicity, number of siblings, survivors’ education, and mothers’ occupation; half of the survivors of childhood brain tumors had mothers who were housewives compared to 37 and 13% of childhood leukemia survivors and healthy children, respectively. Thirteen of the survivors of childhood brain tumors attended special education whereas none of the childhood leukemia survivors and healthy children did so.

**Table 1:** Demographic characteristic of survivors of childhood brain tumor, survivors of childhood leukemia and healthy controls

Characteristic	Childhood brain tumor survivors N (%)	Childhood leukemia survivors N (%)	Healthy control N (%)	*p*-value
Age at study entry, years				
6–10.9	15 (39.5)	16 (42.1)	16 (42.1)	1.000
11–18.9	23 (60.5)	22 (57.9)	22 (57.9)	
Gender				
Male	24 (63.2)	24 (63.2)	24 (63.2)	1.000
Female	14 (36.8)	14 (36.8)	14 (36.8)	
Ethnicity				
Malay	31 (81.6)	27 (71.1)	19 (50)	< 0.005
Chinese	6 (15.8)	1 (2.6)	3 (7.9)	
Indian	1 (2.6)	8 (21.1)	16 (42.1)	
Others	0 (0)	2 (5.3)	0 (0)	
Education				
Primary	15 (39.5)	21 (55.3)	18 (47.4)	< 0.005
Secondary	9 (23.7)	15 (39.5)	18 (47.4)	
Tertiary	0 (0)	1 (2.6)	2 (5.3)	
Special education	13 (34.2)	0 (0)	0 (0)	
None	1 (2.6)	1 (2.6)	0 (0)	
Parents marital status				
Married	37 (97.4)	33 (86.8)	36 (94.7)	0.181
Single parent	1 (2.6)	5 (13.2)	2 (5.3)	
Father’s education level				
No formal education	1 (2.6)	0 (0)	0 (0)	0.760
Primary	6 (15.8)	5 (13.2)	3 (8.3)	
Secondary	20 (52.6)	21 (55.3)	20 (55.6)	
Tertiary	11 (28.9)	12 (31.6)	13 (36.1)	
Father’s occupation				
Office work	7 (18.4)	4 (10.5)	2 (5.6)	0.133
Field work	8 (21.1)	14 (36.8)	15 (41.7)	
Professional	4 (10.5)	6 (15.8)	10 (27.8)	
Others	16 (42.1)	13 (34.2)	8 (22.2)	
Not working	3 (7.9)	1 (2.6)	1 (2.6)	
Mother’s education level				
No formal education	1 (2.6)	0 (0)	0 (0)	0.501
Primary	6 (15.8)	3 (8.1)	5 (13.2)	
Secondary	18 (47.4)	21 (56.8)	15 (39.5)	
Tertiary	13 (34.2)	13 (35.1)	18 (47.4)	
Mother’s occupation				
Office work	8 (21.1)	6(16.2)	7 (18.4)	0.023
Field work	2 (5.3)	1 (2.7)	2 (5.3)	
Professional	5 (13.2)	9 (24.3)	10 (26.3)	
Others	4 (10.5)	7 (18.9)	14 (36.8)	
Housewife	19 (50)	14 (37.8)	5 (13.2)	
Family monthly income				
< MYR 1000	2 (5.3)	0 (0)	1 (2.6)	
MYR 1001–3000	14 (36.8)	10 (26.3)	8 (21.1)	0.328
MYR 3001–5000	8 (21.1)	17 (44.7)	13 (34.2)	
MYR 5001–7000	4 (10.5)	2 (5.3)	2 (5.3)	
MYR 7001–9000	3 (7.9)	3 (7.9)	2 (5.3)	
> MYR 9000	7 (18.4)	6 (15.8)	12 (31.6)	
Number of siblings				
None	5 (13.2)	4 (10.5)	1 (2.6)	< 0.005
1	0 (0)	1 (2.6)	13 (34.2)	
2–5	22 (57.9)	32 (84.2)	24 (63.2)	
> 5	11 (28.9)	1 (2.6)	0 (0)	
Siblings with chronic illness/disability				
Yes	1 (2.6)	0 (0)	0 (0)	0.330
No	37 (97.4)	38 (100)	38 (100)	

*N* number, *MYR* Ringgit Malaysia; analyses using chi square test

**Table 2:** Clinical characteristics of survivors of childhood brain tumor

Characteristics	Mean (± SD)
Age at diagnosis, year	7.2 (3.6)
Age at study entry, year	12.5 (3.7)
Time from end of treatment to study, year	5.5 (3.9)
	N (%)
Pathology	
Medulloblastoma	14 (36.8)
Germ Cell Tumor	10 (26.3)
Craniopharygioma	5 (13.2)
Glioma	2 (5.3)
PNET	2 (5.3)
Others	5 (13.2)
Tumour location	
Supratentorial	21 (55.3)
Infratentorial	17 (44.7)
Treatment	
Tumor excision only	2 (5.3)
Tumor excision + radiotherapy	6 (15.8)
Tumor excision + radiotherapy + chemotherapy	20 (52.6)
Tumor excision + chemotherapy	3 (7.9)
Biopsy + radiotherapy + chemotherapy	2 (5.3)
Biopsy + radiotherapy	2 (5.3)
Chemotherapy + radiotherapy	2 (5.3)
Radiotherapy only	1 (0.9)
Complications from disease and treatment	N (%)
Hydrocephalus	
Yes	24 (63.2)
No	14 (36.8)
Shunt	
Yes	23 (60.5)
No	15 (39.5)
Intracranial infection after diagnosis (ventriculitis)	
Yes	2 (5.3)
No	36 (94.7)
Relapse	
Yes	6 (15.8)
No	32 (84.2)
Disability (Physical, Hearing and Visual)	
Yes	28 (73.7)
No	10 (26.3)
Type of disability	
Physical impairment	13 (34.2)
Hearing impairment	7 (18.4)
Visual impairment	25 (65.8)
Endocrinopathy	
Yes	19 (50)
No	19 (50)

### CBCL and YSR

The survivors of childhood brain tumors showed statistically significantly worse behavioral outcomes for social problems (*p* = 0.006) and attention problems (*p* = 0.007) compared to age- and gender-matched healthy children. When compared to survivors of childhood leukemia, childhood brain tumor survivors showed statistically significantly worse behavioral outcomes for social problems (*p* = 0.022). There were no statistically significant differences in the behavioral outcomes of survivors of childhood brain tumors compared to the two comparison groups on the three global scales (internalizing, externalizing, and total behavioral problems) and other subscales (anxious, withdrawn/depressed, somatic complaints, thought problems, rule breaking, and aggressive behavior) (Table 3).

**Table 3:** CBCL scores of survivors of childhood brain tumor vs survivors of childhood leukemia vs healthy controls

Scales	Childhood brain tumor survivors (*n* = 38) Median (IQR)	Childhood leukemia survivors (*n* = 38) Median (IQR)	Healthy children (*n* = 38) Median (IQR)	*p*-value
Main scales				
Internalizing score	57.5 (50.0–65.5)	53.0 (46.8–63.5)	53.0 (41.0–59.3)	0.061
Externalizing score	52.5 (47.0–58.3)	51.0 (44.0–59.0)	51.5 (42.5–57.5)	0.767
Total score	56.0 (49.8–62.0)	53.0 (44.8–61.0)	52.0 (41.8–57.3)	0.076
Subscales				
Anxious	52.0 (50.8–62.3)	52.0 (51.0–62.3)	51.5 (50.0–60.0)	0.319
Withdrawn/depressed	56.5 (52.0–63.0)	52.5 (50.0–63.0)	53.5 (50.0–58.5)	0.175
Somatic complaints	56.5 (51.0–70.0)	53.0 (50.0–58.8)	53.0 (50.0–61.8)	0.127
Social problems	58.5 (53.0–66.0)	53.5 (51.0–58.5)	52.5 (50.0–58.5)	0.012*
Thought problems	54.0 (50.0–61.0)	52.5 (50.0–58.0)	50.0 (50.0–56.8)	0.168
Attention problems	57.0 (52.0–62.5)	53.0 (51.8–57.0)	54.5 (51.0–57.0	0.024**
Rule breaking behaviour	52.0 (51.0–54.0)	51.0 (50.0–55.5)	51.0 (50.0–53.3)	0.398
Aggressive behavior	53.5 (50.8–57.3)	53.5 (50.0–61.0)	52.0 (50.0–60.0)	0.965

*N* number, *IQR* interquartile range (25th centile –75th centile); analysis using Kruskal-Wallis test

*significant difference is between brain tumor survivors and healthy children and leukemia survivors (Mann-Whitney U test)

**significant difference is between brain tumor survivors and healthy children (Mann-Whitney U test)

statistically significant with *p*-value <0.05

Survivors with either physical, hearing, and/or visual disabilities had poor total behavioral problems (*p* = 0.015), externalizing problems (*p* = 0.006), social problems (*p* = 0.005), rule breaking behavior (*p* = 0.025), and aggressive behavior (*p* = 0.004). In the subgroup analyses, survivors of childhood brain tumors with physical disabilities showed statistically significantly worse outcomes for total behavioral problems (*p* = 0.036), social problems (*p* = 0.001), and attention problems (*p* = 0.028). Survivors with visual impairment showed poor outcomes in total behavioral problems (*p* = 0.019), internalizing problems (*p* = 0.036), and social problems (*p* = 0.010). There was no statistically significant association between hearing impairment and behavioral outcomes in the survivors (Table 4). Statistically significantly worse behavioral outcomes for social problems (*p* = 0.001) were observed in survivors who attended special education compared to those in primary education (Table 5).

**Table 4:** Association between disability and CBCL scoring in survivors of childhood brain tumor

			Disability (Physical disability, visual impairment and/or hearing impairment)			Physical disability			Visual Impairment			Hearing Impairment		
			Yes *n* = 28 Median (IQR)	No *n* = 10 Median (IQR)	*p*-value	Yes *n* = 13 Median (IQR)	No *n* = 25 Median (IQR)	*p*-value	Yes *n* = 25 Median (IQR)	No *n* = 13 Median (IQR)	*p*-value	Yes *n* = 7 Median (IQR)	No *n* = 31 Median (IQR)	*p*-value
CBCL	Main Scales	Internalizing score	58.0 (52.5–67.5)	52.0 (47.5–59.0)	0.061	58.0 (55.5–69.5)	57.0 (49.0–63.0)	0.109	58.0 (55.5–67.5)	52.0 (47.0–61.0)	**0.036**	58.0 (46.0–68.0)	57.0 (50.0–65.0)	0.970
		Externalizing score	55.0 (49.5–59.0)	47.0 (43.7–51.0)	**0.006**	52.0 (47.0–59.0)	53.0 (47.0–57.5)	0.537	55.0 (48.0–59.0)	49.0 (47.0–55.5)	0.175	55.0 (49.0–63.0)	52.0 (47.0–58.0)	0.282
		Total score	58.5 (53.0–63.5)	50.0 (45.5–53.8)	**0.015**	59.0 (53.5–68.0)	52.0 (47.0–60.5)	**0.036**	59.0 (53.5–62.0)	50.0 (45.5–56.5)	**0.019**	59.0 (49.0–67.0)	56.0 (50.0–62.0)	0.678
	Subscales	Anxious	52.0 (51–64.8)	52.5 (50.0–58.3)	0.524	52.0 (51.0–64.5)	52.0 (50.0–61.0)	0.367	52.0 (51.0–64.5)	52.0 (50.0–60.0)	0.419	51.0 (51.0–58.0)	52.0 (50.0–63.0)	0.819
		Withdrawn/Depressed	57.5 (53.3–63.0)	53.0 (50.0–63.8)	0.404	60.0 (53.0–63.0)	54.0 (52.0–64.5)	0.506	58.0 (53.0–64.5)	54.0 (51.0–59.5)	0.227	59.0 (53.0–66.0)	56.0 (52.0–63.0)	0.649
		Somatic complaints	61.0 (50.8–70.0)	53.0 (51.0–55.3)	0.150	64.0 (51.5–75.0)	54.0 (51.0–69.0)	0.149	61.0 (53.0–70.0)	53.0 (50.5–55.5)	0.065	57.0 (50.0–74.0)	56.0 (53.0–70.0)	1.000
		Social problems	61.0 (56.3–67.5)	52.0 (50.0–58.3)	**0.005**	66.0 (59.0–70.0)	56.0 (51.0–61.5)	**0.001**	61.0 (56.6–67)	53.0 (50.0–59.5)	**0.010**	61.0 (58.0–68.0)	58.0 (53.0–66.0)	0.396
		Thought problems	55.0 (51.0–64.0)	52.0 (50.0–56.5)	0.122	58.0 (50.0–68.0)	54.0 (50.0–58.5)	0.242	55.0 (50.0–64.0)	54.0 (50.0–58.5)	0.522	51.0 (50.0–59.0)	54.0 (50.0–61.0)	0.620
		Attention problems	59.0 (53.3–64.3)	53.0 (51.0–60.3)	0.100	59.0 (56.5–66.5)	54.0 (52.0–61.5)	**0.028**	59.0 (54.5–62.0)	53.0 (51.0–64.5)	0.130	57.0 (53.0–65.0)	57.0 (52.0–62.0)	0.706
		Rule breaking behavior	53.0 (51.0–54.0)	51.0 (50.0–52.0)	**0.025**	53.0 (50.0–54.0)	52.0 (51.0–54.0)	0.719	53.0 (50.5–54.0)	51.0 (51.0–52.0)	0.216	53.0 (50.0–54.0)	52.0 (51.0–54.0)	0.565
		Aggressive behavior	55.0 (51.0–59.0)	50.0 (50.0–52.5)	**0.004**	53.0 (51.0–61.0)	54.0 (50.0–57.0)	0.418	55.0 (51.0–58.5)	51.0 (50.0–57.0)	0.181	53.0 (52.0–64.0)	52.0 (50.0–57.0)	0.223

*n* number, *IQR* interquartile range (25th centile –75th centile); analysis using Mann-Whitney U test

statistically significant with *p*-value <0.05

**Table 5:** Association between survivors’ of childhood brain tumor education level and CBCL scores

Survivors’ education level			Primary education *n* = 15 Median (IQR)	Secondary education *n* = 9 Median (IQR)	No formal education *n* = 1	Special education *n* = 13 Median (IQR)	*p*-value
CBCL	Main Scales	Internalizing score	54.0 (48.0–65.0)	58.0 (54.5–60.5)	69.0	58.0 (51.0–69.0)	0.242
		Externalizing score	51.0 (47.0–56.0)	52.0 (43.5–58.0)	44.0	59.0 (49.5–59.5)	0.159
		Total score	53.0 (46.0–59.0)	56.0 (49.5–60.0)	62.0	59.0 (52.0–68.0)	0.150
	Subscales	Anxious	52.0 (50.0–62.0)	52.0 (50.5–59.5)	57.0	55.0 (51.0–64.5)	0.783
		Withdrawn/Depressed	54.0 (50.0–66.0)	54.0 (52.5–61.5)	63.0	58.0 (55.0–61.0)	0.596
		Somatic complaints	55.0 (51.0–61.0)	64.0 (53.0–70.0)	78.0	61.0 (50.0–74.0)	0.233
		Social problems	55.0 (50.0–58.0)	56.0 (52.0–56.0)	69.0	63.0 (60.0–69.0)	0.006*
		Thought problems	54.0 (50.0–58.0)	55.0 (50.5–65.0)	50.0	55.0 (50.5–67.0)	0.416
		Attention problems	55.0 (52.0–61.0)	53.0 (52.0–59.0)	59.0	65.0 (58.0–67.0)	0.050
		Rule breaking behavior	52.0 (51.0–53.0)	51.0 (50.0–54.0)	50.0	53.0 (50.5–54.0)	0.537
		Aggressive behavior	51.0 (50.0–55.0)	52.0 (50.0–58.0)	50.0	57.0 (51.5–63.0)	0.072

n: number, value presented as median (25th centile – 75th centile), analysis using Kruskal-Wallis test

*significant difference is between primary education and special education (Mann-Whitney U test)

statistically significant with *p*-value <0.05

Fathers’ occupation was found to be associated with the CBCL parental reports. However, the results were too variable. Statistically significantly worse behavioral outcomes for thought problems (*p* = 0.026) and rule breaking behavior (*p* = 0.035) were observed in survivors whose fathers worked in the fields compared to those whose fathers performed office work. Upon comparison with the fathers who were professionals, survivors with fathers who worked in the fields showed statistically significantly worse outcomes for attention problems (*p* = 0.010) and rule breaking behavior (*p* = 0.008). Conversely, compared to survivors whose fathers were in the “other” occupation group, survivors with fathers who worked in the fields showed statistically significant problems in rule breaking behavior (*p* = 0.015) (Table 6). Significant findings were also observed in survivors whose fathers did not work compared to fathers who were professionals for total behavioral problems (*p* = 0.032), attention problems (*p* = 0.031), and rule breaking behaviors (*p* = 0.026). Compared to survivors of fathers in the “other” occupation group, survivors of fathers who did not work also demonstrated significantly worse behavioral outcomes for total behavioral problems (*p* = 0.010), thought problems (*p* = 0.014), and attention problems (*p* = 0.016). Isolated poor attention problems (*p* = 0.013) were observed in survivors whose fathers performed office work compared to fathers who were professionals (Table 6). Age at diagnosis, gender, ethnicity, parents’ marital status, parents’ education level, mothers’ occupation, total family income, and number of siblings were not found to be associated with poor behavioral outcomes in our study. Similarly, tumor location, mode of treatment, presence of hydrocephalus, presence of intracranial shunt, history of intracranial infection (ventriculitis) after diagnosis, disease relapse, and presence of endocrinopathy were not found to influence behavioral outcomes in childhood brain tumor survivors.

**Table 6:** Association between father’s occupation and CBCL scores of survivors of childhood brain tumor

Father’s occupation	Not working *n* = 3 Median (IQR)	Office work *n* = 7 Median (IQR)	Field work *n* = 8 Median (IQR)	Professionals *n* = 4 Median (IQR)	Others *n* = 16 Median (IQR)	*p*-value
Main Scale						
Internalizing score	68.0 (59.0–68.0)	67.0 (50.0–70.0)	61.0 (54.8–65.0)	54.5 (46.5–67.8)	54.0 (48.5–58.0)	0.077
Externalizing score	58.0 (55.0–58.0)	56.0 (44.0–59.0)	53.0 (51.0–61.5)	47.5 (44.0–49.5)	50.5 (46.3–57.5)	0.122
Total score	68.0 (61.0–68.0)	59.0 (53.0–65.0)	60.0 (53.8–65.8)	50.5 (46.8–58.0)	51.0 (48.3–58.8)	0.029*
Subscales						
Anxious	56.0 (55.0–56.0)	57.0 (50.0–66.0)	59.0 (52.0–63.5)	53.0 (50.8–70.3)	51.0 (50.0–52.0)	0.124
Withdrawn/depressed	60.0 (60.0–60.0)	63.0 (54.0–68.0)	55.0 (50.0–61.3)	52.5 (50.5–60.5)	56.5 (52.3–62.3)	0.341
Somatic complaints	74.0 (50.0–74.4)	61.0 (53.0–76.0)	57.5 (53.0–70.0)	59.5 (51.3–68.5)	53.0 (50.3–63.3)	0.438
Social problems	68.0 (66.0–68.0)	62.0 (53.0–69.0)	60.5 (55.5–67.5)	53.0 (51.5–65.8)	58.0 (51.5–60.8)	0.132
Thought problems	66.0 (64.0–66.0)	50.0 (50.0–51.0)	57.0 (52.8–60.3)	56.0 (50.3–64.8)	54.0 (50.0–57.3)	0.038**
Attention problems	66.0 (62.0–66.0)	59.0 (53.0–62.0)	61.0 (55.3–70.0)	51.5 (51.0–52.0)	57.0 (52.3–60.5)	**0.011**^**§**^
Rule breaking behavior	54.0 (54.0–54.0)	51.0 (50.0–53.0)	53.5 (53.0–54.8)	51.0 (50.3–51.8)	51.0 (50.0–53.4)	**0.019**^**¶**^
Aggressive behavior	57.0 (56.0–57.0)	55.0 (50.0–61.0)	53.0 (51.0–62.5)	50.5 (50.0–51.0)	53.5 (50.0–57.8)	0.174

n: number, value presented as median (25th centile – 75th centile), analyses using Kruskal-Wallis test

Mann-Whitney U test

*Significant difference is between professionals and not working fathers and between others and not working fathers

**Significant difference is between office workers and field workers and between others and not working fathers

^§^Significant difference is between professionals and office workers; professionals and field workers; professionals and not working fathers, and between those not working and others

^¶^Significant difference is between field workers and office workers; between professionals and field workers; between field workers and others and between professionals and not working fathers

statistically significant with *p*-value <0.05

We found strong correlation between CBCL parent reports and YSR in internalizing problems (Spearman’s coefficient = 0.701, *p* value = 0.001) and total behavioral problems (Spearman’s coefficient = 0.629, *p* value = 0.004). Moderate correlation was found between CBCL parent reports and YSR in externalizing problems (Spearman’s coefficient = 0.419, *p* value = 0.074).

## Discussion

In this study, we found that survivors of childhood brain tumors have more social and attention problems compared to unrelated healthy children and more social problems compared to survivors of childhood leukemia. Social problems among survivors of childhood brain tumors have also been documented in previous studies [6, 8–10, 14, 18, 19]. A few factors may contribute to these findings. Parents who are overprotective may limit the involvement of survivors in family or social activities due to fear of rejection. This could prevent interpersonal skills development and lead to decreased self-confidence and worsened social isolation among survivors [20]. Furthermore, post-traumatic syndrome disorder, which may have occurred during the diagnosis of the brain tumor, and the experience of stress or a traumatic condition during treatment, may continue to affect the survivor for some time. Consequently, the survivors could experience low self-esteem and poor self-concept [21].

Demographic, disease, and/or treatment-related factors can influence behavioral outcomes in survivors of childhood brain tumors. We found that survivors of childhood brain tumors with a combination of physical, visual, and/or hearing impairments or any one of these components reported poor behavioral outcomes. Survivors with a physical disability were found to have significant total behavior, social, and attention problems. Survivors with a visual impairment had significant total behavior, internalizing, and social problems. A spectrum of motor deficit could occur in survivors of brain tumors including hemiplegia, leg muscle weakness, spasticity, and abnormal gait due to the tumor itself or the corresponding treatment. Motor deficits contribute to declines in functional status and health-related quality of life in survivors [22]. Decreased function in gross motor skills, particularly balancing and running speed, has been observed in posterior fossa tumor survivors [23]. Survivors of childhood brain tumors were also found to have reduced muscle strength and fitness similar to those found in individuals over 60 years old [24]. All this could lead to limitations in physical performance and restricted participation in home, social, and educational activities, eventually causing poor social functioning, social isolation, and low self-esteem [24].

Carpentieri SC et al. reported that tumor location was a risk factor for behavioral problems among survivors of childhood brain tumors however, Poggi Get et al. found no significant differences between CBCL scores and site or type of tumor [10, 12]. Age at the time of diagnosis and age when chemotherapy and radiation therapy were commenced were not reported to be significantly related to internalizing or externalizing maladaptive behaviors [6, 9, 13]. Holmquist LA et al. reported that vincristine, cytoxan, cisplatinum, and/or etoposide drugs were associated with late onset emotional and behavioral problems with internalizing behaviors being the most prevalent and significant in survivors of brain tumors [6]. Although radiation therapy has been frequently associated with poorer behavioral and social outcomes in survivors of childhood brain tumors, some researchers reported that the type and total dose of radiation therapy and extension of the radiotherapy field did not have any impact on parents’ ratings of behavioral problems [6, 9, 10]. Likewise, the magnitude of tumor resection and insertion of a shunt for hydrocephalus were not associated with childhood brain tumor survivors’ behavior [6, 9]. Nevertheless, most previous studies assessed heterogenous groups of survivors of childhood brain tumors, which may obscure patterns in behavioral outcomes related to specific locations or treatment modalities. Poretti A et al. studied outcomes in craniopharyngioma survivors and reported that both YSR and parent-rated CBCL results showed clinically significant scores in total and internalizing problems with social problems as the most affected subscale [25]. Dolson EP et al. found that craniopharyngioma survivors who had extensive tumor resection manifested baseline internalizing and externalizing problems before conformal irradiation [26]. In this subgroup of survivors, the presence of cerebrospinal fluid shunting, Ommaya reservoir for cyst drainage, diabetes insipidus, and lower pre-irradiation growth hormone levels were predictors for worse behavioral outcomes after conformal radiation therapy [26]. In another study, patients with high-risk treatment, those with posterior fossa syndrome, and females were found to have greater withdrawn/depressive and social problems [27]. We did not find any significant association between behavioral outcome and location of tumour, mode of treatment, presence of hydrocephalus, presence of intracranial shunt, history of intracranial infection after diagnosis, disease relapse and presence of endocrinopathy in our study population.

We found an association between survivors of childhood brain tumors and their education level. Those survivors who attended special education were found to have more social problems compared to survivors in primary education. This is explained by the intellectual, physical, visual, or hearing impairments in the survivors following the treatment received. In general, survivors whose fathers worked in the fields and those who did not work showed poor behavioral outcomes. Fathers who worked in the fields and were unemployed may have a lower education level and might have difficulty in understanding the disease, be anxious, or lack a coping mechanism.

We did not identify significant differences in behavioral problems between survivors of childhood brain tumors and leukemia survivors on the global scales and most of the subscales. This supports the findings of Carpentieri SC et al. and Holmquist LA et al. who reported that survivors of childhood brain tumors did not have more behavioral problems than survivors of non-CNS tumors [6, 12]. However, we must cautiously interpret these findings because parents may underreport behavioral problems in their children. Some parents may be overprotective of their children who survived a brain tumor because they perceive them as very vulnerable. Overprotective parents and their difficulties with encouraging autonomy in survivors of brain tumors have been documented previously [28]. Nevertheless, the lack of knowledge on long-term emotional and behavioral problems and its urgency from the parents’ perspective may also contribute to underreporting [28]. Thus, survivors of childhood brain tumors should be regularly assessed for possible emotional and behavioral problems.

### Strengths and limitations

Many previous studies compared the findings on survivors of brain tumors with normative values but rarely used a comparison group. A shortcoming of only comparing to normative data is overestimating the rate of behavioral problems in survivors. Our study recruited two comparison groups; thus, the results are expected to have a higher validity. However, this study has a few limitations that should be considered when interpreting the results. The study population was small, and the participants had heterogenous diagnoses. Given the small prevalence of childhood brain tumors, this is a challenge despite the involvement of two centers in this study. A nationwide collaboration would help minimize this limitation. Furthermore, a longitudinal study would be more predictive and accurate in revealing behavioral changes that occur over time, particularly any emotional stress patterns in survivors.

## Conclusions

Our study findings show that survivors of childhood brain tumors exhibited significant behavioral problems, specifically social and attention problems. Physical disabilities, visual impairment, survivor’s education level, and father’s occupation were associated with poor behavioral outcomes.

## Data Availability

The datasets used and/or analyzed during the current study are available from the corresponding author on reasonable request.

## Abbreviations

ALL: Acute lymphoblastic leukemia
CBCL: Child Behavior Checklist
CNS: Central nervous system
SES: Socioeconomic status
YSR: Youth Self-Report
